# Impact of low sperm competition on male reproductive trait allometries in a bush-cricket

**DOI:** 10.1186/s12862-019-1514-0

**Published:** 2019-10-11

**Authors:** Lennart Winkler, Leon M. Kirch, Klaus Reinhold, Steven A. Ramm

**Affiliations:** Department of Evolutionary Biology, Bielefeld University, Morgenbreede 45, 33615 Bielefeld, Germany

**Keywords:** Allometry, Sexual selection, Sperm competition, Testes, Sperm removal, Spermatophore gland, Subgenital plate, One-size-fits-all hypothesis

## Abstract

**Background:**

Studying reproductive trait allometries can help to understand optimal male investment strategies under sexual selection. In promiscuous mating systems, studies across several taxa suggest that testes allometry is usually positive, presumably due to strong selection on sperm numbers through intense sperm competition. Here, we investigated testes allometry in a bush-cricket species, *Metaplastes ornatus*, in which females mate promiscuously, but where sperm removal behaviour by males likely drastically reduces realised sperm competition level.

**Results:**

As hypothesised, we found evidence for negative testes allometry and hence a fundamentally different male investment strategy compared to species under intense sperm competition. In addition, the mean relative testes size of *M. ornatus* was small compared to other species of bush-crickets. Surprisingly, the spermatophore gland, a potential alternative trait that males could invest in instead of testes, also did not show positive allometry, but was approximately isometric. We further observed the expected pattern of negative allometry for the male morphological structure responsible for sperm removal in this species, the subgenital plate, supporting the one-size-fits-all hypothesis for intromittent genitalia.

**Conclusion:**

Our findings suggest that the evolution of sperm removal behaviour in *M. ornatus* was a key adaptation for avoiding sperm competition, with important consequences for reproductive trait allometries. Nevertheless, they also imply that it does not pay for larger males to invest disproportionately in nuptial gift production in this species.

## Background

The evolution of male reproductive traits, such as bigger testes size, is usually driven by selection on the ability to achieve fertilization success. These selection pressures often originate from high levels of sperm competition in mating systems where females mate with multiple males during a single reproductive cycle [1–4].

In addition to often mating multiply, female insects, in particular, are often able to store and maintain sperm in specially adapted spermathecae and other reproductive organs, further promoting high levels of sperm competition [5–8]. This has led to a variety of male adaptations and strategies in order to achieve fertilization success, and these can broadly be considered as arising either from selection on mechanisms for pre-emption of stored sperm (from other males) or selection on mechanisms to prevent pre-emption of (own) stored sperm, i.e. as offensive and defensive sperm competition traits, respectively [1, 5, 9]. In both categories, adaptations can exist on a morphological as well as on a behavioural level. Known defensive adaptations include for instance prolonged copulation durations as found in the lovebug *Plecia nearctica*, which mates for up to 56 h [10]; extensive post-copulatory mate guarding, for example in the firebug *Pyrrhocoris apterus* [11]; mating plugs that prevent the female from remating like those deposited by male checkerspot butterflies, *Euphydryas chalcedona* [12]; and copulating pairs that hide from the swarm to avoid male takeovers, as observed in some Nematocera flies [10].

By contrast, offensive pre-emption mechanisms to maximise fertilization success focus either on the mechanical displacement of previously deposited sperm from rival males or other means to ensure either the numerical or qualitative precedence of one’s own sperm. Examples of mechanical displacement can be found in male crickets, damselflies and dragonflies [13–15], who use their genitalia to physically remove or compact the sperm of the female’s prior mates before they inseminate her with their own sperm, with similar methods found also in longicorn beetles *Psacothea hilaris* [16] and the earwig *Euborellia plebeja* [17]. By contrast, in other insect groups sperm predominance can be achieved by the production of longer sperm [18], more sperm [19] or a higher sperm viability [20]. However, these latter adaptations to sperm competition all share the necessity to make a greater investment in sperm production in order to increase fertilization success. As in many other animal taxa (e.g. [21–24]), one way this investment can be, and frequently is, manifested in male insects is through increased testes size [18, 25–27] (reviewed in [8, 28, 29]), though this measure alone does not necessarily capture all aspects of increased investment in sperm production [30].

A common method to investigate the selection pressures on investment in certain traits is to describe how body size and the compared trait relate to each other. This scaling relationship between body size and a specific trait is called static allometry [31]. Static allometries are typically described by a power law [32] and a log-transformation of the axes achieves a linear relationship [33]. This makes it possible to use the slope as a description of the body–trait relationship. If *an allometric slope is* greater than one, a positive allometric relationship is observed, indicating relatively bigger traits in bigger animals. The reverse is true for *slopes smaller than* one, where a negative allometric relationship describes relatively smaller traits in bigger animals. In the case of *a slope equal to* one, the trait size increases in proportion to body size, i.e. exhibits isometry [34]. A common finding for many sexually selected traits is that these exhibit positive allometry [35], but this is far from a universal pattern and for any given species, the patterns of allometry we can expect will reflect specific investment trade-offs that determine net fitness returns [36].

In the presence of strong sperm competition, testes are expected to exhibit positive allometry, because larger individuals can invest proportionally more in testes size than small ones. Positive allometry evolves when viability, as probability for mating period survival, increases with body size in a saturating manner. Individuals should then benefit more from investment in sexual traits, since the margin for increasing their viability by growing larger is limited [31]. Past studies have not provided clear results on the evolution of positive allometry under sperm competition and different species often differ significantly in their allometry [37, 38]. However, these studies generally indicate positive static allometry in testes size in species with potentially high levels of sperm competition (e.g. [39–41]). Positive testes allometry has also recently been shown in the bush-cricket species *Poecilimon veluchianus* [41]. Whereas the positive allometry of testes size in *P. veluchianus* is consistent with the promiscuous mating system and expected high levels of sperm competition in this species [41], we here sought to investigate testes allometry in a second bush-cricket that appears to differ markedly in this regard. Specifically, although females often mate multiply, male bush-crickets of the species *Metaplastes ornatus* perform a specialised sperm removal behaviour [42], which radically alters their expected level of sperm competition. Before transferring their own spermatophore to the female which contains both sperm and a nutritious nuptial gift [2], *M. ornatus* males perform repeated bouts of copulatory behaviour with their mate, during which they introduce their highly modified subgenital plate into the female genital tract to remove sperm of rivals [42]. Sperm removal thus reduces the level of sperm competition to a significant degree, with on average only about 15% of the previous male’s sperm typically remaining in the female genital tract after sperm removal [42]. Moreover, males invest more time in sperm removal behaviour with non-virgin mates [43]. We can therefore predict that sperm removal drastically alters selection on sperm production and thereby testes allometry, since under a low realised level of sperm competition there is likely reduced selection on producing high sperm numbers.

A second male reproductive trait that may be under sexual selection in *M. ornatus* is the spermatophore gland responsible for spermatophore production. The spermatophore contains both sperm (in the ampulla) and the nuptial gift (spermatophylax) containing water, proteins and some nutrients [44], and is transferred to the female during mating. The quality of this nuptial gift likely affects the number of eggs the female will lay, as this has been shown in other bush-cricket species [44]. Additionally, a large nuptial gift prevents the female from consuming the sperm before they are transferred to the female reproductive tract [45], and comparative evidence suggests that investment in larger spermatophores may help prevent females from remating [46]. Hence, spermatophore glands are directly linked to male fertilization success and might therefore in general be expected to exhibit positive allometry [39]. Since sperm competition in *M. ornatus* should be at a low level due to sperm removal [42], we further hypothesized that larger males might invest proportionally more in their spermatophore gland (i.e. nuptial gift production) than in testes size (i.e. sperm production). Some benefits of the nuptial gift, like the number of eggs the female will lay [44], should be beneficial for males independently of the level of sperm competition. Overall, this should lead to a shallow allometric slope for testes and a steeper slope of spermatophore gland allometry. Moreover, by comparing two related bush-cricket species (i.e. our data for *M. ornatus* with the previous report for *P. veluchianus* [41]; see also [47]) with potentially extreme differences in sperm competition level, we can indirectly infer the impact of variation in this selection pressure on the allometry of essential male reproductive traits.

Finally, we investigated the allometry of a third trait for which we would predict negative allometry. The subgenital plate is the highly-derived male structure that actually contacts with the female genital tract during sperm removal behaviour to effect the removal of sperm, and as such we expect that it is a genital trait likely to conform to the one-size-fits-all hypothesis [48]. Specifically, this hypothesis is thought to apply when a trait – like genitalia – must fit a specific structure and is therefore not under directional selection, but rather stabilizing selection [39]. Organs that are meant to be introduced into the female genital tract should thus not deviate too much from the mean size of the female genital tract, hence they should not change much with body size [39, 48, 49]. The subgenital plate of *M. ornatus* is such an organ: being essential for sperm removal [42], it must fit well into the female genital tract. We therefore additionally hypothesized that subgenital plate allometry is negative with a slope close to zero.

In addition to investigating the allometries of three male reproductive traits as a function of hindleg length, we also included a measure of condition, to test if short-term fluctuation in mass explains additional variance. We measured condition as a proxy using the residuals from a linear regression of body mass on hindleg length.

Overall, by estimating allometric slopes for these three key reproductive traits – testes, spermatophore glands and genitalia – for a species likely to experience low realised sperm competition levels due to sperm removal, we hope that data from *M. ornatus* can contribute to a wider understanding of how variation in sperm competition level impacts on male reproductive investment strategies and trait allometries.

## Results

**Fig. 1:**
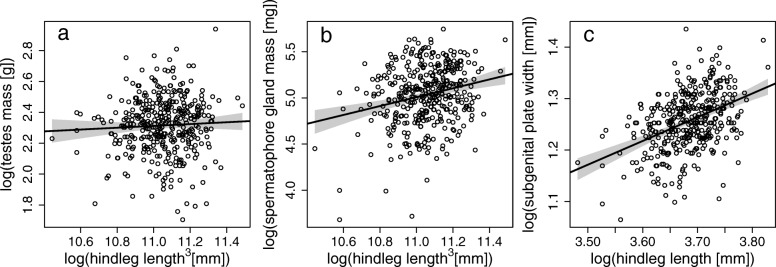
Trait allometries of log testes mass (**a**), log spermatophore gland mass (**b**) and log subgenital plate width (**c**), with log hindleg length on x-axes. Black line represents OLS and grey area 95% CIs

Beginning with the testes, we found that the allometric slope of 0.234 was significantly greater than zero (*p* = 0.0005) (Table 1, Fig. 1a), but significantly smaller than one (*p* < 0.0001), although the slope estimated through RMA was somewhat higher and significantly greater that one (see Additional file 1: Table S1). Overall, our data suggest that mean testes mass is about 1.33% of the mean body mass in *M. ornatus*.

**Table 1:** Allometries for testes weight, spermatophore gland mass and subgenital plate width in *Metaplastes ornatus*. OLS models using hindleg length (to the power of three for testes and spermatophore gland models), with population origin as fixed factor (common slope). Additionally, the *p* value testing for a difference of the slope to one is given. df = 379

Variable	Estimate	Std. error	t value	p (H_0_:0)	p (H_0_:1)
log testes mass [mg]	0.234	0.066	3.519	0.0005	< 0.00001
log spermatophore gland mass [mg]	0.778	0.111	6.996	< 0.00001	0.047
log subgenital plate width [mm]	0.398	0.057	6.942	< 0.00001	< 0.00001

To examine the other major gland responsible for ejaculate production, we next investigated allometry of the spermatophore gland. Spermatophore gland mass exhibits a slightly negative allometry. The estimated slope of 0.778 was significantly different from zero (Table 1, Fig. 1b) and (marginally) smaller than one (*p* = 0.047), whereas the RMA analysis would have suggested positive allometry for this trait (see Additional file 1: Table S1).

Finally, we tested the hypothesized negative allometry of the male intromittent genitalia. The subgenital plate allometry was indeed negative according to the OLS analysis, with an estimated slope of 0.398 (Table 1, Fig. 1c). Again, the RMA slope estimate is higher, and in this case more indicative of isometry (see Additional file 1: Table S1).

All OLS slopes computed including condition as an additional explanatory variable were somewhat steeper than the equivalent analysis without this covariate (Table 2). Condition is significantly, positively related with testes mass (Table 2). Also, subgenital plate width increases significantly with increasing condition (Table 2).

**Table 2:** Results of OLS regression for all three traits, including population origin as a fixed effect and condition as a covariate. df = 379, except for spermatophore gland model 377

Trait	Variable	Estimate	Std. error	t value	*p* value
log testes mass [mg]	log hindleg length^3^ [mm]	0.258	0.065	3.958	< 0.00009
	Condition	0.330	0.076	4.337	< 0.00002
log spermatophore gland mass [mg]	log hindleg length^3^ [mm]	0.898	0.078	11.540	< 0.00001
	Condition	18.987	6.310	3.009	0.028
	log hindleg length^3^ [mm] x condition	−1.556	0.572	−2.723	0.007
log subgenital plate width [mm]	log hindleg length [mm]	0.414	0.057	7.301	< 0.00001
	Condition	0.075	0.022	3.395	0.0008

In the spermatophore gland model, hindleg length and condition interact significantly, with decreasing effect of hindleg length/condition on the correlation with spermatophore gland weight, as condition/hindleg length increase (see Table 2). This means, that larger animals increased spermatophore gland mass less if there was a further increase in their condition, compared to small individuals or that animals in overall good condition increased gland mass less even given comparably big body size.

## Discussion

While testes allometry is found to be positive in many species with high levels of sperm competition [31, 39–41], relaxed sperm competition might favour the evolution of alternative investment strategies, since allometric patterns are ultimately the result of selection acting simultaneously on different traits that affect fitness. In the case of *M. ornatus*, we examined three such traits that could affect male reproductive success and for which different optimal allometric scaling might be predicted: testes mass, spermatophore gland mass and the size of the subgenital plate (which effects sperm removal in this species). Whilst the RMA slope we estimated for testes mass was indicative of (weak) positive allometry, the poor correlation between hindleg length and testes mass (*r* = 0.05) means we prefer to interpret the OLS slope estimates. Here, the data clearly indicate a shallow allometric slope of 0.234. This negative testes allometry contrasts with the positive testes allometry found in *P. veluchianus* [41]. Likely the reason for the different resource investment strategies in these two bush-cricket species is sperm removal performed by males of *M. ornatus* but not *P. veluchianus*. Sperm removal has the potential to very substantially reduce the level of sperm competition in *M. ornatus* [42], suggesting investment in sperm production is less crucial for male fitness if sperm competition is relaxed, presumably favouring resource allocation to other traits.

Additionally, there was a difference in the mean proportion of testes mass on body mass between the two species. While in *M. ornatus* the testes made up 1.33% of the total body mass, in *P. veluchianus* this was 2.21% [41]. This difference in relative investment into testes mass is in line with the hypothesised lower investment into sperm production in *M. ornatus* that is due to the reduced sperm competition. When comparing the relative investment of *M. ornatus* into testes mass with other species of bush-crickets (Tettigoniidae) it is important to account for differences in body size, because interspecific allometry likely differs from isometry [50]. Therefore, we calculated the “relative testes size” (RTS) as observed- over expected testes size (after [50]). We find that RTS for *M. ornatus* was with 0.896 smaller than expected, comparing it with 21 other species of Tettigoniidae (after [51]). However, it is also important to note that the link between sperm competition, testes mass and sperm numbers is also somewhat unclear in Tettigoniidae: although across species there is a strong positive correlation between degree of polyandry and testes mass [51], there is also a negative correlation between testes mass and ejaculate size as estimated by ampulla mass [46, 51]. This could indicate that testes size in bush-crickets is predominantly under selection via mating rate rather than sperm competition per se, but it could also mean that testes size becomes less important if chemical or mechanical properties of the ampulla (that led to its larger size) lower the benefit of an increased number of sperm per ejaculate.

Similarly to the reduced investment in sperm competition indicated by lower allometric slopes of testes mass in the present study, a recent paper reports that the testes allometry of two species of mice differed in accordance with different sperm competition intensities [52]. Here the polygynous/promiscuous mating system of the mound-building mouse (*Mus spicilegus*) might lead to enhanced sperm competition [52].

We further hypothesized that *M. ornatus* males might rather invest in the spermatophore gland and by that the nuptial gift as an alternative route to maximising reproductive success. However, our findings provide only limited evidence that there is positive allometry for spermatophore gland weight: whilst the RMA slope was positive, the OLS slope clearly instead suggests negative allometry with a slope slightly smaller than one. One complication here is that the spermatophore gland is in fact comprised of two tissues, the much larger one that produces the spermatophylax material and the smaller one that produces the material that builds the ampulla. Nonetheless, the allometric slope of spermatophore gland is steeper than that of testes, with non-overlapping confidence intervals. Hence the hypothesis that reduced sperm competition in this species leads to investment into the nuptial gift rather than sperm production is supported by these data, but spermatophore gland size alone cannot fully explain where *M. ornatus* invests resources potentially not used for sperm production.

Given the absence of positive allometry for spermatophore gland size, it seems like the benefits of increasing investment into the spermatophore gland might saturate. A potential alternative investment route that we did not explore here is that males might use resources not invested into sperm production rather in sperm removal behaviour. An alternative explanation is that not only the benefit of testes, but also of spermatophore gland investment decreases with lower sperm competition. Sakaluk [2] hypothesised in an intraspecific study of nuptial gift feeding, that nuptial gift size in bush-crickets might increase with larger (numerical) sperm competition, but comparative data would appear to indicate the opposite and support our initial expectation of a potential trade-off: bush-crickets with greater investment in testes tend to make lower investment in spermatophore glands, and vice versa [51].

As expected, based on the one-size-fits-all hypothesis [48], we found negative allometry for the sub-genital plate. The width of the subgenital plate should be optimized to fit into the female genital tract. Therefore, it should be under stabilizing selection and should not change with body size. Nevertheless, the slope was significantly larger than zero (and actually even larger than the slope for testes allometry). However, past studies also did not always find a zero slope for comparable traits (see e.g. [49]). Anyway, there are several (non-exclusive) evolutionary explanations outlined in the following for the slope not to be zero and even a positive allometry would be supported by the allometric slope estimated via RMA (see Additional file 1: Table S1). If larger males prefer larger females (and vice versa), assortative mating [53] could lead to an allometric slope different to zero. As larger males mate with larger females in this scenario, they might need larger subgenital plates. It is also possible that the effectiveness of the subgenital plate is dependent on its width. If larger width is more effective, there would be selection on this trait, increasing the allometric slope. Both explanations should result in a slope larger than zero and a deviation from the one-size-fits-all hypothesis. Further studies are needed to test if these hypotheses could be true.

Condition can play an important role in allometric relationships, as all three allometric slopes were steeper if condition was considered. Although this did not change any of the broad conclusions above about these slopes, it suggests an additional influence of resource availability on allometric scaling, an effect which was particularly pronounced for the spermatophore gland. Additional resources of males in good condition are rather invested into spermatophore gland, than sperm production.

That condition was predicted by subgenital plate size might lead to this trait being an honest signal of male quality and thus this trait could be subject to cryptic female choice on ‘good genes’ [54]. Here cryptic female choice could play a role in the evolution of genital morphology [55] (reviewed in [56]). This could also give an additional explanation to why the allometric slope of subgenital plate width was different to zero. If there is cryptic female choice on ‘good genes’ based on the subgenital plate width, larger males would benefit from further investment in this trait. This could lead to the allometric slope being larger than expected, even though the one-size-fits-all hypothesis might still be generally applicable here. Further research is needed to show whether females prefer males with bigger subgenital plates (pre- or post-copulatory sexual selection) and whether subgenital plate width predicts male reproductive success.

Additionally, there was a significant interaction of hindleg length and condition in spermatophore gland allometry. This might mean that hindleg length/condition becomes less influential on the allometric slope as condition/hindleg length increases. That males that are big and/or in good condition seem to invest less in the spermatophore gland, could be a sign that the payoff of this investment saturates, explaining the negative allometry we found in this study. Further studies are needed to test if this hypothesis holds true.

## Conclusions

This investigation of allometry in *M. ornatus* enables us to shed some light on how different mating systems can alter the allometric slope. Our results likely demonstrate that male trait allometries can be substantially affected by the evolution of sperm removal behaviour and its consequent impact on realised sperm competition levels. This study also provides a novel, indirect reinforcement of how sperm removal does indeed lower the level of sperm competition in *M. ornatus*. In addition, we show that the one-size-fits-all hypothesis likely is met for the subgenital plate size in *M. ornatus*. Nevertheless, the presented allometric slopes found were steeper than predicted by theory, which leaves room for alternative explanations and needs further investigation. Overall, comparing the allometries of related species with different mating systems can help to understand the different trade-offs that shaped the evolution of these species.

## Methods

### Data collection

We collected 441 adult males from three different populations of *Metaplastes ornatus* [57] in central Greece in June 2017. Aiming for large variation in body size [58], the bush-crickets were collected from populations located at different altitudes (*n* = 175 individuals from a ‘low’ altitude population ca. 5 km North of Vitoli (DD: 38.9788 lat., 22.0084 long.) (460 m asl); *n* = 171 individuals from a ‘middle’ altitude population close to Paleokastro (DD: 38.9877 lat., 21.9017 long.) (790 m asl); and *n* = 95 individuals from a ‘high’ altitude population above Rovoliari (DD: 39.0085 lat., 21.9976 long.) (990 m asl)). To control for the unknown timing of the last mating of males before collection from the field, all collected individuals were kept for 48 h after capture in a 16x26x15.5 cm plastic cage with up to 20 other males with ad libitum supply of Judas tree (*Cercis siliquastrum*) leaves but no access to females, to prevent mating and equalise ejaculate reserves. After these two days, males were weighed (Sartorius Quintix digital balance (d = 0.0001 g)) and prepared for dissection by removing the head. Afterwards, hindleg femur, tibia and total hindleg length were measured (Whitwhorth digital calliper (d = 0.1 mm)) as an indicator of overall body size. A subset of the hindleg measurements were repeated after ca. 30 min to estimate repeatability. The dissected testes and spermatophore glands were weighed immediately to prevent loss through evaporation (Sartorius quintix digital balance (d = 0.0001 g)). Each testis was weighed separately (repeatability ICC = 0.45, *n* = 382) and the combined spermatophore gland mass was weighed. All raw morphological data is provided in Additional file 2.

### Statistical analysis

Upon processing for dissection, all individuals that were found to contain endoparasites or had a missing leg were excluded from the final dataset. Final sample sizes were *n* = 156 for ‘low’, *n* = 149 for ‘middle’ and *n* = 78 for ‘high’ altitude population (total *n* = 383).

Soma mass was calculated by subtracting testes mass, when testes mass was the dependent variable. Likewise, spermatophore gland mass was subtracted from total body mass, when gland mass was the dependent variable. Dimensions were standardised where necessary by taking hindleg length measurements to the power of three. Allometry was explored using log transformed data (log here always refers to the natural logarithm). Linear models (OLS) were calculated using R (v3.1.1, in RStudio v1.0.153) [59] and the packages ‘lme4’ (v1.1–10) [60] and ‘lmerTest’ (v2.0–30) [61]. Population origin was included in models as a fixed effect. As expected, the higher the sampling altitude, the lower was the mean body mass (data not shown). Reduced major axis models were calculated using R packages ‘smatr’ [62].

While some papers suggest to use the reduced major axis technique (RMA, type 2 regression) to estimate allometric slopes (e.g. [63]), in contrast to OLS (type 1 regression), it is still a matter of some debate which method represents the better statistical approach (see [64]) and there has been experimental support for OLS to be used in the context of allometry [65, 66]. Furthermore, for low correlation coefficients like in this dataset (testes = 0.05; spermatophore gland = 0.23; subgenital plate = 0.42) the estimated RMA slopes are highly inflated [66]. We therefore focus on the OLS slopes in the presented analyses as the more conservative approach, but for completeness report also results using RMA in the supplementary information (see Additional file 1: Table S1). Nevertheless, irrespective of whether we used OLS or RMA, the broad conclusions comparing *M. ornatus* allometries to *P. veluchianus* remain qualitatively unchanged (see Results).

Hindleg length was used to determine animal size. Because hindleg length does not capture the animal’s current condition, in an additional analysis a condition score was added to the OLS models as a covariate (see Table 2). The condition score consists of the residuals of the log-log regression of body mass on hindleg length (data not shown). It was also tested if condition interacts with hindleg length. However, this interaction was only significant in the spermatophore gland model (see Table 2) and was hence dropped for the other models.

In contrast to other studies (e.g. [41]), not the hindleg femur length, but rather total hindleg length was used in the present analyses, because we found hindleg length had lower measurement error compared to femur and tibia length (data not shown). Based on repeated measurements for a subset (*n* = 318) of the data, hindleg length was measured with high repeatability (ICC = 0.951).

## Supplementary information

**Additional file 1: Table S1.** Allometries for testes mass, spermatophore gland mass and subgenital plate width. RMA models using hindleg length giving the 95% CI as lower/upper limits. Additionally, the *p* value testing for a difference of the slope to one is given. Df = 379.

**Additional file 2.** Raw data.

## Data Availability

The data set supporting the results of this article is available as Additional file 2.

## Abbreviations

ICC: Intra-class correlation
OLS: Ordinary least squares
RMA: Reduced major axis
RTS: Relative testes size
