# Supplementary material for: Impact of low sperm competition on male reproductive trait allometries in a bush-cricket
Source: BMC Evol Biol. 2019 Oct 11;19:185. doi: 10.1186/s12862-019-1514-0 (PMC6788016; doi:10.1186/s12862-019-1514-0)
Supplement: Supplementary file 1 — Additional file 1: Table S1. Allometries for testes mass, spermatophore gland mass and subgenital plate width. RMA models using hindleg length giving the 95% CI as lower/upper limits. Additionally, the p value testing for a difference of the slope to one is given. Df = 379. [file 12862_2019_1514_MOESM1_ESM.pdf]

Additional file 1

Table S1: Allometries for testes weight, spermatophore gland weight and subgenital plate width. RMA models using hindleg length giving the 95% CI as lower/upper limits. Additionally, the p value testing for a difference of the slope to 1 is given. Df= 379.

Variable	Estimate	Lower limit	Upper limit	p value (diff. 1)
Testes	1.267	1.135	1.413	0.0002
Spermatophore gland	2.170	1.948	2.417	<0.00001
Subgenital plate	1.149	1.038	1.271	0.007
